# An umbrella review navigating the nationwide burden of hepatitis B virus infection in Ethiopia: A call for action on vaccination, safe blood, and infection prevention

**DOI:** 10.1371/journal.pone.0352169

**Published:** 2026-06-22

**Authors:** Abel Desalegn Demeke, Aboma Tsegaye, Alem Bayable, Takla Tamir, Biazin Yenealem Mekuriaw, Behailu Taye Gebremeskele, Ababo Demeke, Mesfin Abebe, Abraham Dessie Gessesse, Delelegn Emwodew Yehualashet, Habtamu Gebrie, Endris Seid Amede

**Affiliations:** 1 Department of Nursing, College of Medicine and Health Sciences, Dilla University, Dilla, Ethiopia; 2 Department of Psychiatry, College of Medicine and Health Sciences, Dilla University, Dilla, Ethiopia; 3 Department of Medical Laboratory Science, College of Medicine and Health Sciences, Dilla University, Dilla, Ethiopia; 4 Department of Midwifery, College of Medicine and Health Sciences, Dilla University, Dilla, Ethiopia; 5 Department of Pediatrics and Child Health Nursing, College of Health Sciences, Woldia University, Woldia, Ethiopia; 6 School of Public Health, College of Medicine and Health Sciences, Dilla University, Dilla, Ethiopia; University of Rwanda College of Medicine and Health Sciences, RWANDA

## Abstract

**Background:**

The Hepatitis B virus (HBV) is one of the major causes of viral hepatitis that may persist and lead to complications, including cirrhosis, hepatic decompensation, and liver cancer. Numerous systematic reviews and meta-analysis have been conducted on HBV infection among people in Ethiopia, showing inconsistent findings. This umbrella review aimed to assess the pooled prevalence of HBV infection and its associated factors among people in Ethiopia.

**Methods:**

All published and unpublished systematic review and meta-analysis studies were searched on databases such as PubMed, Scopus, HINARI and Google Scholar. A measurement tool to assess systematic reviews was used for critical appraisal of the included studies. STATA software version 17 was used to conduct the analysis. I^2^ test were used to determine heterogeneity, whereas funnel plots and Egger’s regression tests were used to assess publication bias. To pool the prevalence of HBV infection, a random-effects model was used and presented using a forest plot.

**Results:**

Twelve systematic reviews and meta-analysis with 997,264 participants were included in this umbrella review. The pooled prevalence of the HBV infection among people in Ethiopia was 5.78% (95% CI: 5.33, 6.23). Being male, having multiple sexual partners, having a history of abortion, having body tattoos, having a history of tooth extraction, sharing sharp material, and linked to health system such as having a history of hospital admission, and blood transfusion were significant risk factors associated with HBV infection among people in Ethiopia.

**Conclusion:**

This study highlights that the prevalence of HBV infection among people in Ethiopia was relatively moderate endemicity as per WHO classification. By synthesizing evidence of prior review, this study provides specific up-to-date information for clinicians and policymakers to design evidence-based public health strategies and guide future research that enable the prevention, control, and eliminate infection of HBV.

**PROSPERO Registration**: CRD420251160887

## Introduction

### Background

Hepatitis is defined as a disease characterized by an inflammation of the liver, which is caused by infectious viruses and non-infectious agents [1]. Viral hepatitis is the leading infectious cause of death globally [2]. Among the five types of hepatitis virus (A, B, C, D, and E), hepatitis B virus (HBV) is one of the major causes of viral hepatitis that may persist and lead to complications including cirrhosis, hepatic decompensation, and liver cancer [1,3]. People may be infected by HBV through different modes of transmission, such as direct contact with infected blood or body secretion from an infected mother to child, sexual contact with an infected person, or inappropriate medical procedures [4,5]. Similarly, as evidenced a different risk factors that contribute to HBV infection were having an HBV infected family member, multiple sexual partners, abortion history, blood transfusion, family history of chronic liver disease, exposure to body fluid, tattooing, and sharp needle injury [6–8].

According to the evidence synthesized from 187 countries by the WHO, around 254 million people are living with HBV infection, and 1.2 million were newly infected by HBV worldwide in 2022. The statistics were depicted: around 1.3 million people died from viral hepatitis, of which 83% was caused by HBV, and there was an increment of hepatitis B mortality by 34% from 2019 to 2022. In Africa, over 60 million estimated people were living with chronic hepatitis B [9]. A recent systematic review and meta-analysis conducted found the prevalence of HBV infection among healthcare workers in Africa to be 17.2% [10] and pregnant women in East Africa to be 6% [11].

According to Worldometer’s elaboration of the most recent United Nations data, Ethiopia’s population as of Saturday, May 9, 2026, is 138,308,684 [12]. The federal, regional, and municipal governments oversee the nation’s highly decentralized health system, which is primarily dependent on a community-based Health Extension Program (HEP) and functions under a three-tier delivery system with the goal of increasing access to primary care [13]. The pentavalent vaccine, which is typically administered at 6, 10, and 14 weeks, contains the hepatitis B vaccine as part of Ethiopia’s Expanded Program on Immunization (EPI). In a significant move to prevent mother-to-child transmission (MTCT), the Ministry of Health formally started a national Hepatitis B Birth Dose (HepB BD) vaccine program in late 2025, with plans to roll it out nationwide in 2026 [14]. National and international legislation underline that blood donation must be voluntary and unpaid [15].

To meet the 2030 sustainable development goal of eliminating viral hepatitis, we are left with only five years. In Ethiopia various systematic reviews and meta-analysis have been conducted [7,8,16–24] (EPHI and MOH, [Unpublished]) and found inconsistent findings, with the pooled prevalence of HBV infection ranging from 4.7% in pregnant women [19] to 7.4% [21] in the general population. These variations highlight a gap in understanding the current up-to-date burden of HBV infection among populations in Ethiopia. As far as our searching in PubMed, Google Scholar, and the International Prospective Register of Systematic Reviews (PROSPERO), there are neither umbrella reviews nor ongoing published protocols examining HBV virus infection in Ethiopia.

Systematic reviews and meta-analyses are common methods for synthesizing information of outstanding quality. To provide a thorough understanding of a field of study, new techniques known as umbrella reviews are now being developed that combine results from several systematic reviews and meta-analyses rather than primary studies [25]. In order to help physicians and policymakers create evidence-based public health strategies and direct future research that will allow them to prevent, control, and eradicate HBV infection, the current umbrella review is crucial. Thus, this umbrella review aimed to assess the pooled prevalence of HBV infection and its associated factors among people in Ethiopia.

## Methods

The planned protocol of this umbrella review was registered in the PROSPERO database and obtained a unique registration ID, CRD420251160887. This study adhered to the Preferred Reporting Items for Systematic Review and Meta-Analysis (PRISMA) umbrella review methodological guidelines [26] (**S1 Table**).

### Databases and search strategy

A literature search was conducted on the following prominent databases: PubMed, Scopus, HINARI, and Google Scholar. Two authors independently (ADD and BY) conducted a search from September 6, 2025, to September 17, 2025, using keywords such as “prevalence,” “seroprevalence,” “Hepatitis B virus,” “HBV,” “Hepatitis B surface antigen,” “HBsAg,” “viral hepatitis,” “Ethiopia,” “systematic review,” and “meta-analysis” combined with the “AND” and “OR” Boolean operators (**Table 1**). The research question with explicit eligibility criteria of this review was formulated through using the Condition, Context, and Population (CoCoPop) framework.

**Table 1:** A searching strategy.

Databases	Search term	Number of article	Search Date
Pubmed	((“hepatitis b virus”[Title/Abstract] OR “HBV”[Title/Abstract] OR “hepatitis b surface antigen”[Title/Abstract] OR “HBsAg”[Title/Abstract] OR “viral hepatitis”[Title/Abstract]) AND “Ethiopia”[Title/Abstract]) AND (meta-analysis[Filter] OR systematicreview[Filter])	20	9/6/2025
HINARI	((TitleCombined:(Hepatitis B virus)) OR (HBV) OR (hepatitis b surface antigen)) AND (HBsAg) AND OR (Systematic review)) AND (meta analysis)	217	9/7/2025
Google Scholar	(“prevalence” OR “seroprevalence”) AND (hepatitis b virus” OR “HBV” OR “hepatitis b surface antigen” OR “HBsAg” OR “viral hepatitis”) AND (“Ethiopia”)	58	9/12/2025
SCOPUS	(TITLE-ABS-KEY (“Prevalence” OR “seroprevalence”) AND TITLE-ABS-KEY (“hepatitis b virus” OR “HBV” OR “hepatitis b surface antigen” OR “HBsAg” OR “viral hepatitis”) AND TITLE-ABS-KEY (“Ethiopia”) AND TITLE-ABS-KEY (“Systematic review” OR “meta-analysis”))	21	9/17/2025

### Inclusion and exclusion criteria

All published and unpublished systematic reviews with meta-analysis conducted through utilizing observational study design (cross-sectional, cohort, and case-control) among people in Ethiopia with no exclusion criteria on age, sex, race, or health status and reporting prevalence or risk factors of HBV infection without restriction on publication year and language were included. On the contrary, studies that did not assess prevalence or risk factors of HBV infection, narrative reviews, primary studies, systematic reviews without meta-analysis, expert opinions, case reports, editorials, correspondence, scoping reviews, and methodological studies were excluded.

In these studies, unpublished studies were described as studies that had not been sourced from peer-reviewed journals [27].

### Study selection process

A retrieved article was imported into EndNote version X8 software; following this, duplicates were removed. Subsequently, two authors (ADD and TT) independently screened the title and abstract for predefined inclusion and exclusion criteria. Following this, the full-text article was evaluated, and those that met the inclusion criteria were included in the final analysis. Any disagreements were resolved through discussion with the impartial mediator of the third author (AB).

### Data extraction

Two authors (ADD and ADG) conducted extraction of data from the included SRMA studies using a standardized Microsoft Excel spreadsheet. The following information was extracted: author name, publication year, aim of the study, number and study design of primary studies, sample size, participants, quality assessment tool, and prevalence of HBV infection with confidence interval. Issues of discrepancies in data extraction process were resolved by the mediator of the third author (BTG).

### Measurements of outcomes

The primary objective of this umbrella review was to determine the overall prevalence of HBV infection in Ethiopia. The prevalence was calculated by dividing the number of people who were infected with HBV by the total number of people in the study, then multiplying the result by 100. The second objective of this study was to identify the factors associated with HBV infection in Ethiopia. The association between the variables and HBV infection was estimated by the pooled odds ratios (POR).

### Assessment of methodological quality

The new update of “A Measurement Tool to Assess Systematic Reviews” (AMSTAR 2) was used to evaluate the quality of the included SRMA. It enables a standardized approach to examine different aspects of a review, such as its methodology, transparency, and risk of bias (RoB). The AMSTAR 2 had 16 items with three possible options: “yes,” “partial yes,” or “no.” Of them, 6 items, such as registration of protocol, adequacy of search strategy, assessment of risk of bias (RoB), methods of meta-analysis appropriateness, use of RoB during interpretation, and assessment of publication bias, were considered as critical domains, while the remaining 10 were considered as non-critical. As a result, the quality of the study is classified as ‘high’ (0 or 1 non-critical weakness), ‘moderate’ (> 1 non-critical weakness but 0 critical flaws), ‘low’ (1 critical flaw with or without non-critical weaknesses), or ‘critically low’ (> 1 critical flaw with or without non-critical weaknesses) [28,29]. Two authors (ADD and ABD) independently assessed the methodological quality of included SRMA studies; any discrepancies were resolved through discussion or with the third author (HG) before final decisions were made.

### Assessment of primary study overlap

Although there is no universally accepted method for assessing the overlap of primary studies across included SRMA studies, we have utilized Corrected Covered Area and visually mapped the overlap using the Graphical Representation of Overlap for Overview’s (GROOVE) tool, in which this dual method enhances identification and management of overlap, depicting that evidence generated from the study remains accurate, robust, and free from bias [30]. CCA is used to quantify the degree of study overlap by calculating the proportion of shared studies using the following formula.


$$\begin{array}{c} CCA=\frac{N-r}{(r\times c)-r}\;, \end{array}$$

Whereas, N is total number of primary studies that appeared in the review; r, number of rows (total number of unique primary studies); c is the number of included reviews (number of columns); CCA is the corrected covered area

The value are categorized as slight (0–5%), moderate (6–10%), high (11–15%), and very high (>15%) overlap [31]. The GROOVE tool used to assess a detailed CCA for each possible pair of SRMA through graphical representation of these results [32] and is freely available at http://doi.org/10.17605/OSF.IO/U2MS4 and https://es.cochrane.org/es/groovetool.

### Data synthesis and analysis

The commercial software STATA/MP version 16 was used to carry out a statistical analysis. The Pooled prevalence of hepatitis B infection and its associated factors was estimated by a random effects model through utilizing the method of DerSimonian and Laird [33]. The inverse variance (I^2^) index test was used to assess heterogeneity across the studies, in which the results of <50%, 50–75%, and >75% suggested low, moderate, and high, respectively [34]. The factors variable was considered to be significantly associated with HBV infection if the 95% CI of the pooled odds ratio did not include 1. The publication bias was measured by the funnel plot and Egger’s regression test, through observation and P-value, respectively [35,36]. Asymmetrical distribution and a P-value < 0.05 on the funnel plot and Egger’s regression test indicate the presence of publication bias, respectively, and are handled by a non-parametric trim and fill analysis [37]. Furthermore, a sensitivity analysis was conducted to identify the influence of a single study on the overall pooled estimate. A P-value < 0.05 with a 95% confidence interval was determined to be statistical significant. Finally, the findings of this umbrella review were presented descriptively, along with tables and figures.

## Result

### Description of study selection process

**Fig 1:**
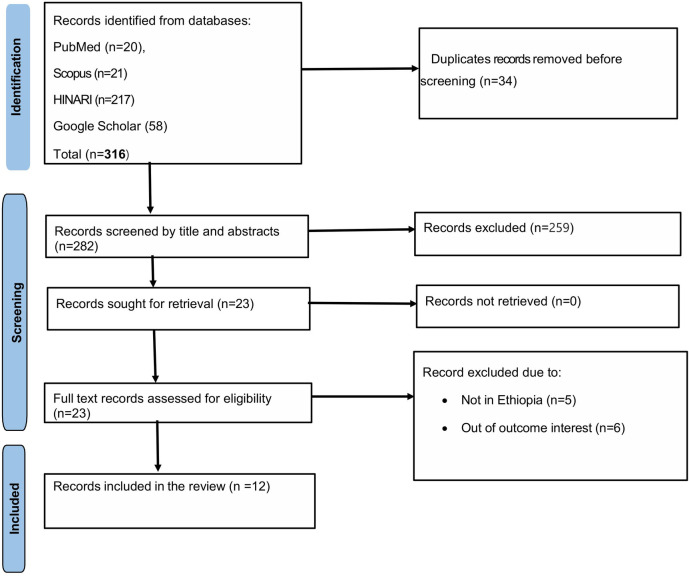
PRISMA flow chart for identification and selection of studies for inclusion in the umbrella review.

The initial search of databases yields a total of 316 records. Of them, 34 records were removed before screening due to duplication. Then, 259 records were removed after a thorough screening of titles and abstracts. Finally, the full text of 23 records was assessed for eligibility, in which 12 of them met the inclusion criteria and were included in this umbrella review [7,8,16–24] (EPHI and MOH, [Unpublished]) (Fig 1).

### Characteristics of included studies

In the current umbrella review we have included twelve SRMA studies, with 997,264 participants. The number of primary studies across the SRMA ranged from 8 [24] to 72 [7] and the sample size ranged from 2,127 [24] to 391,339 [23]. All studies included had reported the tool used to assess the methodological quality, in which the JBI critical appraisal tool was the most commonly used to evaluate methodological quality [7,8,16,17,19,23,24], with three reviews using the Newcastle-Ottawa Scale [18,20,22] and two reviews using the Downs and Black checklist [21] (EPHI and MOH, [unpublished]). Among the studies included in this umbrella review, the protocols of seven SRMA [7,8,16–18,23,24] were registered on the PROSPERO database and reported their unique registration number, but the other five SRMA [19–22] (EPHI and MOH, [unpublished]) failed to provide details of protocol registration. The estimated pooled prevalence of HBV infection ranged from 4.75% [20] to 7.4% [21] among pregnant women and the general population, respectively (**S2 Table**).

### Primary study overlap across the included studies

As shown in **S2 Table**, a total of 406 primary studies were incorporated across 12 included SRMA studies. However, only 200 unique primary studies [38–234] were identified after a critical appraisal of the 12 SRMA studies organized in column-wise and their corresponding primary studies organized in row-wise, which indicates that at least two primary studies appeared in multiple SRMA. Two primary studies were found with maximum repetition in six SRMA, seven primary studies in five SRMA, fourteen primary studies in four SRMA, forty primary studies in three SRMA, and forty-six primary studies in two SRMA. Among the 200 unique primary studies, 91 were found in the single specific individual SRMA (non-overlapped primary study). The overall CCAs were 9.36%, which is a moderate overlap among SRMA. Across the included SRMA studies, we have found 66 total nodes (pairs of reviews); of them, 13 had very high overlap (≥15%), 5 had high overlap (10% to < 15%), 6 had moderate overlap (5% to <10%), and 42 had slight overlap (<5%) (**S3 Table**).

**NOTE:** The discrepancy between the total number of unique primary studies (200) and cited references (197) mentioned above is due to three studies [172,188,212] that had similar citations being used twice in the SRMA done by Belyhun et al [21].

### Methodological quality of included studies

From the included SRMA studies, half of them (6) [7,8,17,18,23,24] were classified as high quality, with 1 SRMA [16] as moderate quality. The remaining 5 SRMA studies [19–22] (EPHI and MOH, [unpublished]) were rated as low quality. However, in this umbrella review, no included SRMA studies were found to be of critically low quality (**S4 Table**).

### Prevalence of hepatitis B virus infection in Ethiopia

**Fig 2:**
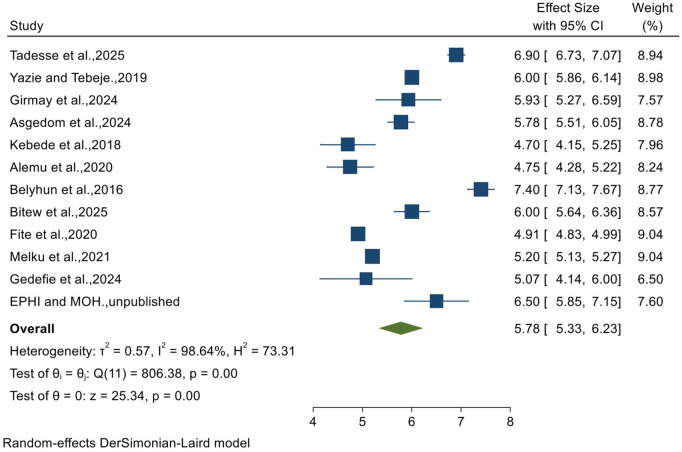
Forest plot showing pooled prevalence of hepatitis B infection among people in Ethiopia.

Based on the random effect model, the overall polled prevalence of hepatitis B virus infection in Ethiopia was 5.78% (95% CI: 5.33, 6.23). According to the findings of our study, a significant high heterogeneity was observed across the twelve studies included in this umbrella review (I^2^ = 98.64, p = 0.000) (Fig 2). As a result, a subgroup analysis was conducted by publication year and study participant.

### Subgroup analysis

**Fig 3:**
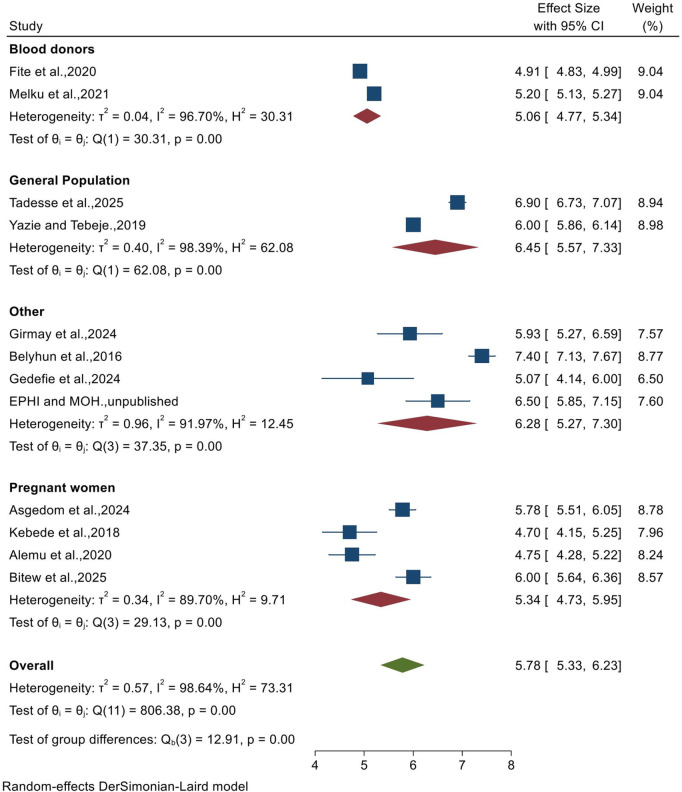
Subgroup analysis by category of study population for prevalence of hepatitis B infection among people in Ethiopia.

The result of subgroup analysis by study population showed that the highest prevalence of HBV infection was found among the general population, 6.45% (95% CI: 5.57, 7.33; I^2^ = 98.39; p = 0.00) and the lowest in blood donors, 5.06% (95% CI: 4.77, 5.34; I^2^ = 96.7%; p = 0.00) (Fig 3).

**Fig 4:**
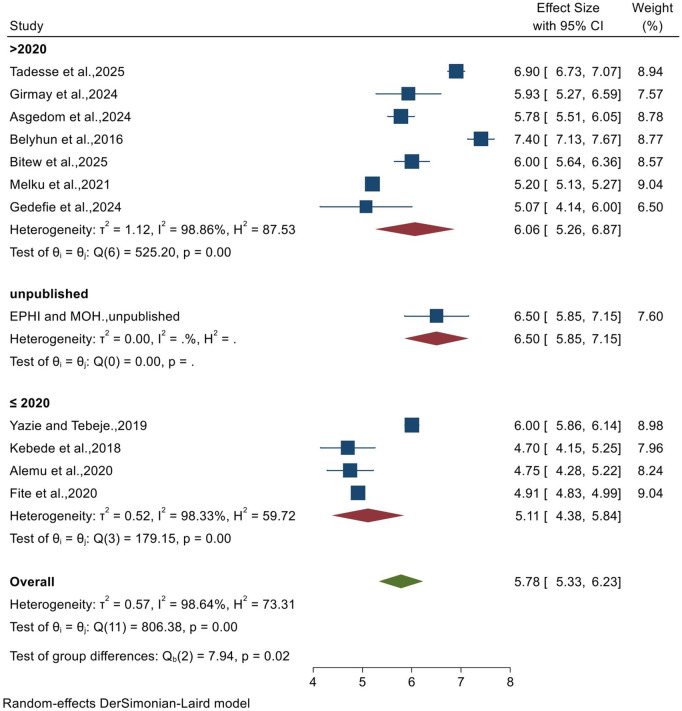
Subgroup analysis by publication year category for prevalence of hepatitis B infection among people in Ethiopia.

In addition, the subgroup analysis conducted by publication year showed that studies published after 2020 reported the highest prevalence of HBV infection rate of 6.06% (95% CI: 5.26, 6.87; I^2^ = 98.86; p = 0.000), whereas the lowest prevalence detected among studies published in 2020 or before was 5.11% (95% CI: 4.38, 5.84; I^2^ = 98.33%; p = 0.00) (Fig 4).

### Publication bias

**Fig 5:**
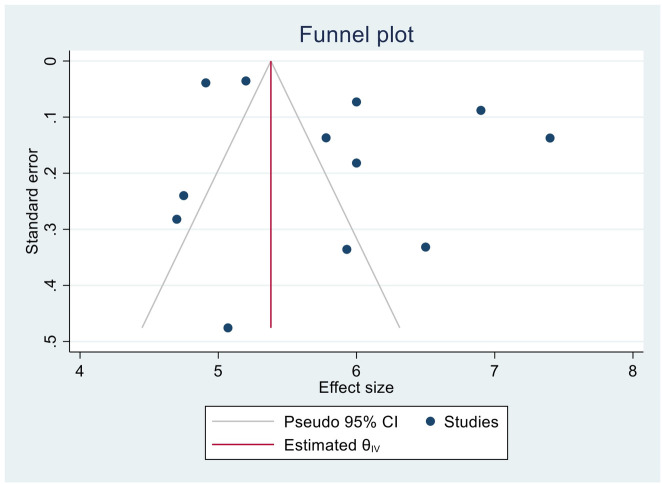
A funnel plot for publication bias for the prevalence of hepatitis B virus infection among people in Ethiopia.

The objective statistical assessment of publication bias by Egger’s regression depicted the absence of publication bias across the included studies (p-value = 0.5622). But the asymmetric distribution shown in the funnel plot indicates the presence of publication bias among the included SRMA (Fig 5). To detect and correct publication bias, a non-parametric trim and fill analysis was conducted. The result showed that no imputed study was observed in the analysis. Effect sizes for observed and imputed are equal (5.776). The similarity of effect size after trim-and-fill analysis indicated the absence of publication bias (**S5 Table**).

### Sensitivity analysis

**Fig 6:**
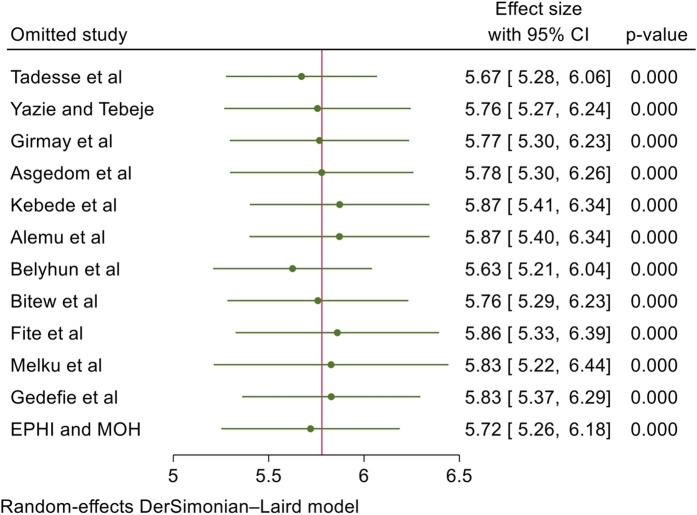
Sensitivity analysis for the prevalence of hepatitis B virus infection among people in Ethiopia.

In the current umbrella review, we have performed a leave-one-out sensitivity analysis to declare the effect of a particular study on the overall polled estimate. The result of sensitivity analysis showed no point estimates of the omitted analysis lie outside the CI of the overall combined estimate. This verified that no single SRMA study had significantly affected the overall polled estimate and confirmed the reliability of the pooled prevalence of HBV infection (Fig 6).

### Factors associated with hepatitis B virus infection

In this umbrella review, variables found as risk factors of HBV infection in at least two SRMA, such as multiple sexual partners, history of abortion, history of tattooing, history of blood transfusion, history of tooth extraction, history of hospitalization, and being male, were taken into account.

### Association between gender and HBV infection

**Fig 7:**
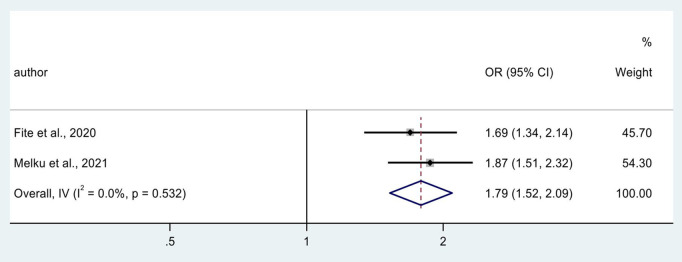
Association of male gender with hepatitis B virus infection.

Two studies [22,23] showed a significant association between male gender and HBV infection. In this review, we identified that the odds of HBV infection were approximately two times higher among males as compared to females (OR = 1.79; 95% CI: 1.52, 2.09; I^2^ = 0.0%; P = 0.532) (Fig 7).

### Association between multiple sexual partners and HBV infection

**Fig 8:**
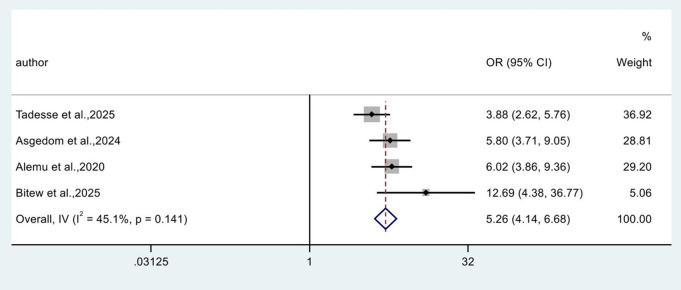
Association of multiple sexual partners with hepatitis B virus infection.

Four SRMA studies [7,8,18,20] had found a significant association between multiple sexual partners and HBV infection. As a result of this umbrella review, the odds of HBV infection were five times higher among individuals who had multiple sexual partners as compared to their counterparts (OR = 5.26; 95% CI: 4.14, 6.68; I^2^ = 45.1%; P = 0.141) (Fig 8).

### Association between abortion history and HBV infection

**Fig 9:**
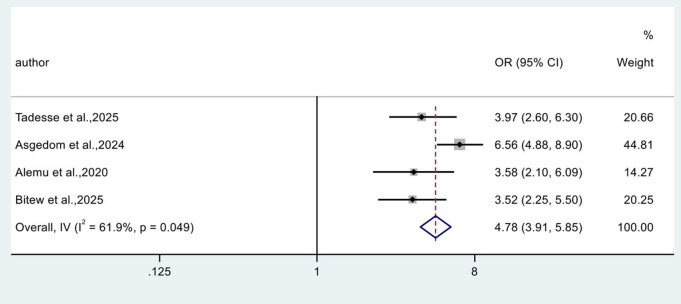
Association of abortion history with hepatitis B virus infection.

Four studies [7,8,18,20] reported a statistically significant association between abortion history and HBV infection. Our pooled analysis found that the odds of HBV infection were approximately five times higher among women who had an abortion history as compared to their counterparts (OR = 4.78; 95% CI: 3.91, 5.85; I^2^ = 61.9%; P = 0.049) (Fig 9).

### Association between body tattoo and HBV infection

**Fig 10:**
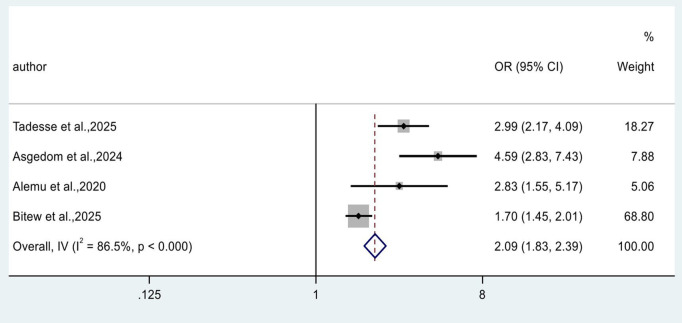
Association of body tattoo with hepatitis B virus infection.

In four included SRMA studies [7,8,18,20] we found a significant association between tattooing and HBV infection. The pooled analysis of our review revealed that the odds of HBV infections were two times higher among individuals who had body tattoos as compared to their counterparts (OR = 2.09; 95% CI: 1.83, 2.39; I^2^ = 86.5%; P < 0.000) (Fig 10).

### Association between blood transfusion and HBV infection

**Fig 11:**
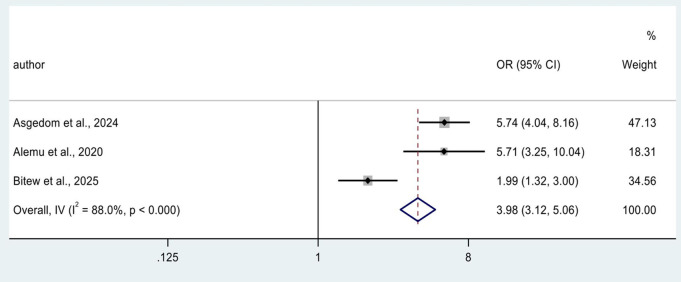
Association of blood transfusion with hepatitis B virus infection.

In three SRMA studies [8,18,20] a significant association was found between blood transfusion and HBV infection. The pooled analysis of this study showed the odds of HBV infection were nearly four times higher among people with a history of blood transfusion as compared to their counterpart (OR = 3.98; 95% CI: 3.12, 5.06; I^2^ = 88.0%; P < 0.000) (Fig 11).

### Association between tooth extraction and HBV infection

**Fig 12:**
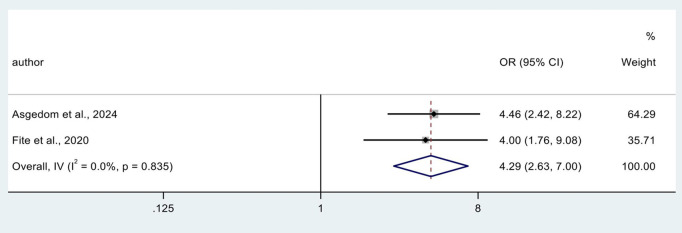
Association of tooth extraction with hepatitis B virus infection.

Two studies reported [18,22] a significant association between tooth extraction and HBV infection. Our result showed that the polled odds ratio of HBV infection was four times higher among individuals with tooth extraction as compared to their counterparts (OR = 4.29; 95% CI: 2.63, 7.00; I^2^ = 0.0%; P < 0.835) (Fig 12).

### Association between sharing sharp material and HBV infection

**Fig 13:**
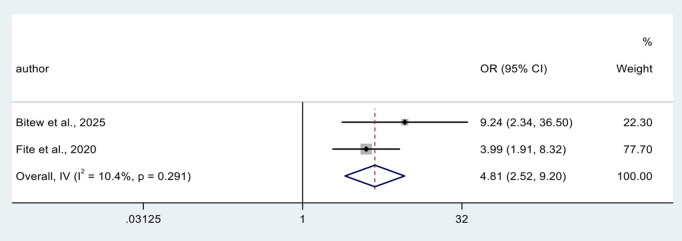
Association of sharing sharp material with hepatitis B virus infection.

Two SRMAs [8,22] explored a significant association between HBV infection and sharing of sharp material. This study revealed that the odds ratio of HBV infection was approximately five times higher among individuals who had shared sharp material as compared to their counterparts (OR = 4.81; 95% CI: 2.52, 9.20; I^2^ = 10.4%; P = 0.291) (Fig 13).

### Association between hospital admission and HBV infection

**Fig 14:**
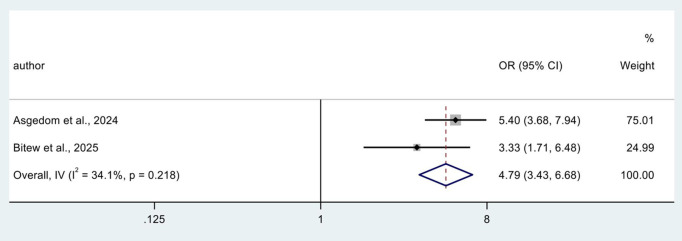
Association of hospital admission with hepatitis B virus infection.

Among the included SRMA studies, two of them [8,18] reported a significant association between hospital admission and HBV infection. Our umbrella review result revealed that the odds of HBV infection were 4.79 times higher among individuals who had a history of hospital admission as compared to their counterparts (OR = 4.79; 95% CI: 3.43, 6.68; I^2^ = 34.1%; P = 0.281) (Fig 14).

## Discussion

In this umbrella review, we set out to determine the nationwide burden of HBV infection in the Ethiopian population. Our analysis revealed that HBV infection prevalence (5.78%) is moderate, per the WHO criteria for endemicity of HBV: ≥  8%, 2–7%, and < 2% were categorized as high, moderate, and low, respectively. The findings of this study highlight pivotal information for evaluating the effectiveness of Ethiopia’s present prevention and control tactics and can be used as a guide to create and carry out efficient public health management initiatives that would help achieve the 2030 elimination target. Our pooled prevalence of HBV infection is similar to that of Ethiopia (5.3%) [235], a systematic review and meta-analysis conducted across four East African countries (6.025%) [236], ten East African countries (6%) [11] and seven West African countries (5%) [237]. However, it is greater than that of Europe (0.6%) [238], China (3%) [239] and India (1.6%) [240] but lower than that of systematic reviews and meta-analysis in Africa (17.2%) [10], Nigeria (9.5%) [241] and Sudan (12.07%) [242]. Similarly, it is lower than the study that reported the wartime prevalence of HBV infection (8.3%) in the Tigray region, Ethiopia [243]

This umbrella review demonstrated significant factors associated with increased risk of HBV infection. The findings suggest that the male gender may be associated with increased odds of HBV infection compared to the female. These findings were similar to previously conducted studies in people with severe mental illness [244], military personnel [245], the general population [246], adults [247] and patients [248]. This might be due to exposure of males to high-risk behaviors such as drug use, unsafe sex, etc. The current study showed that the prevalence of HBV infection has an association with multiple sexual partners. People who have multiple sexual partners are more likely to be infected by HBV infection as compared to those who don’t have multiple sexual partners. This finding is in agreement with a previously conducted study [249,250] The possible reason behind this is that, as evidence depicted, having multiple sexual partners is the main risk factor for sexually transmitted diseases, which play a pivotal role in the transmission of HBV [251].

According to this study, the odds of HBV infection were high among women who had a history of abortion as compared to their counterparts. This finding is in line with a previously conducted systematic review and meta-analysis among pregnant women that assessed associations between prior abortion and HBV infection [252]. This could be due to unsafe abortion increasing the risk of HBV infection. The odds of HBV infection were higher among people with body tattoos. This result agreed with a prior study [253]. Individuals who had a blood transfusion history were nearly four times more likely to be infected by HBV. This is supported by studies in India [254]. This is possible due to the fact that any fluid, such as blood or mucosal contact from an infected person, is one of the ways of transmission for HBV infection.

This study highlights that individuals who had a history of tooth extraction were at higher risk of being infected by HBV. This is consistent with studies in Somali [255]. The possible explanation for this might be due to contamination of dental instruments, poor practice of sterilization, and traditional dental extraction procedures, which play a role in the infection of HBV. In addition, in the present study, a significant association was found between sharing of sharp material and HBV infection. The odds of HBV infection were higher among individuals who share sharp material as compared to their counterparts. This finding is consistent with systematic review and meta-analysis in East Africa [11]. This is possible due to those materials increasing the chance of exposure to infected blood or body fluid. Furthermore, our study revealed that the odds of HBV infection were higher among individuals who had a history of hospital admission as compared to those who had no hospital admission history. The reason behind this might be that health facilities increase the risk of infection by HBV through exposure to body fluid.

### Strength and limitation of the study

The strength of this study is that it serves as a benchmark for future researchers undertaking their interventional and other high-standard studies to reduce the burden of HBV infection. In addition, it strictly followed the PRISMA guideline and comprehensively searched the available literature through different databases. However, the result should be interpreted by taking study limitations into account. We found a high heterogeneity in the pooled prevalence of HBV infection and a moderate overlap of primary studies across the included SRMA studies.

## Conclusions

In this review we found the prevalence of HBV infection among people in Ethiopia was 5.78%, which is moderate endemicity as per WHO classification. Being male; having multiple sexual partners; having a history of abortion; having a body tattoos, history of tooth extraction; sharing sharp material; and factors linked to health-related exposure such as having a history of hospital admission and blood transfusion, were consistently significant factors associated with HBV infection among people in Ethiopia. By synthesizing evidence of prior review, this study provides specific up-to-date information for clinicians and policymakers to design evidence-based public health strategies and guide future research that enables them to prevent, control, and eliminate infection of HBV.

## Supporting information

S1 Table A PRISMA 2020 checklist. (DOCX)

S2 Table Characteristics of the studies included in the umbrella review on the prevalence of hepatitis B infection in Ethiopia. (DOCX)

S3 Table Graphical representation and corrected covered area of overlapped primary studies across included studies in the umbrella review. (XLSX)

S4 Table Quality assessment of included studies using the AMSTAR 2 for assessing quality of include systematic review and meta-analysis in the umbrella review. (DOCX)

S5 Table A trim and fill analysis for publication bias on the prevalence of hepatitis B virus infection among people in Ethiopia. (PDF)

S1 Data (XLSX)

## Abbreviation:

AMSTAR: A measurement tool for assessment of multiple systematic reviews
CI: confidence interval
CCA: Corrected covered area
GROOVE: Graphical Representation of Overlap for Overview’s
HBV: Hepatitis B virus
OR: Odds ratio
PROSPERO: International Prospective Register of Systematic Reviews
PRISMA: Preferred Reporting Items for Systematic Reviews and Meta-Analyses
SRMA: Systematic review and meta-analysis
WHO: World Health Organization.
