# Supplementary material for: An umbrella review navigating the nationwide burden of hepatitis B virus infection in Ethiopia: A call for action on vaccination, safe blood, and infection prevention
Source: PLoS One. 2026 Jun 22;21(6):e0352169. doi: 10.1371/journal.pone.0352169 (PMC13286280; doi:10.1371/journal.pone.0352169)
Supplement: S2 Table — (DOCX) [file pone.0352169.s002.docx]

| Authors | Publication Year | Aim or objective | Number of primary studies with study design | Search Strategy (Electronic databases) | Sample size | Study participants | Information of protocol registration | Quality assessment | Prevalence of HBV (95% CI) |
| --- | --- | --- | --- | --- | --- | --- | --- | --- | --- |
| Tadesse et al., 2025 [[7](file:///C:\Users\abel\Desktop\PLOSE%20Hepatitis\Revision%20hepatitis\Manuscript.docx#_ENREF_7)] | 2025 | To determine the pooled  prevalence and risk factors for HBV infection in Ethiopia | CS = 71  CC = 1 | PubMed, African Journals Online, Google Scholar | 83,058 | General Population | PROSPERO (CRD42024493993) | JBI | 6.9% (95% CI: 6.1, 7.7) |
| Yazie and Tebeje, 2019 [[16](file:///C:\Users\abel\Desktop\PLOSE%20Hepatitis\Revision%20hepatitis\Manuscript.docx#_ENREF_16)] | 2019 | To estimate the  overall pooled prevalence of HBV among the Ethiopian population | CS = 60 | PubMed, Google Scholar, Science Direct, African Index Medicus,  African Journals Online, WHO Afro Library | 106,125 | General Population | PROSPERO (CRD42019131382) | JBI | 6% (95% CI: 5, 6) |
| Girmay et al., 2024 [[17](file:///C:\Users\abel\Desktop\PLOSE%20Hepatitis\Revision%20hepatitis\Manuscript.docx#_ENREF_17)] | 2024 | To determine the pooled prevalence of hepatitis B and  C infections among HCWs in Ethiopia | CS = 18 | PubMed, Cochrane Library, Science Direct, Hinari, African Journals Online | 4,948 | Healthcare workers | PROSPERO (CRD42024527940) | JBI | 5.93% (95% CI: 3.22, 8.63) |
| Asgedom et al., 2024 [[18](file:///C:\Users\abel\Desktop\PLOSE%20Hepatitis\Revision%20hepatitis\Manuscript.docx#_ENREF_18)] | 2024 | To investigate the pooled seroprevalence of hepatitis B virus infection and factors  associated with pregnant women in Ethiopia | CS = 48 | PubMed/Medline, Science Direct, Web of  Science, Google Scholar, Hinari, Cochrane Library | 29,067 | Pregnant women | PROSPERO (CRD42023438522) | NOS | 5.78% (95% CI: 5.14, 6.43) |
| Kebede et al., 2018 [[19](file:///C:\Users\abel\Desktop\PLOSE%20Hepatitis\Revision%20hepatitis\Manuscript.docx#_ENREF_19)] | 2018 | To estimate the overall prevalence of hepatitis B virus infection among  pregnant women in Ethiopia | CS = 17 | PubMed, Popline, Lalicus, Ovid, MedNar,  African Journal Online (AJOL),advanced Google Scholar | 5629 | Pregnant women | NA | JBI | 4.7% (95% CI 4.0–5.4%) |
| Alemu et al., 2020 [[20](file:///C:\Users\abel\Desktop\PLOSE%20Hepatitis\Revision%20hepatitis\Manuscript.docx#_ENREF_20)] | 2020 | To determine the pooled prevalence of HBV and its associated factors in Ethiopia | CS = 23 | Google Scholar, African Online Journal, CINAHL, PubMed | 7,860 | Pregnant women | NA | NOS | 4.75% (95% CI: 4.06, 5.44) |
| Belyhun et al., 2016 [[21](file:///C:\Users\abel\Desktop\PLOSE%20Hepatitis\Revision%20hepatitis\Manuscript.docx#_ENREF_21)] | 2016 | To provide a quantified estimate of the problem as a step toward for a better understanding of the viral hepatitis epidemiology, clinical burden and the situation of human immunodeficiency virus (HIV) co-infection in Ethiopia | CS = 39  PCS = 2  RS = 1 | PubMed, Google scholar, Medline, Web of Science | 36,343 | General population | NA | DAB | 7.4% (95%CI: 6.5, 8.4) |
| Bitew et al., 2025 [[8](file:///C:\Users\abel\Desktop\PLOSE%20Hepatitis\Revision%20hepatitis\Manuscript.docx#_ENREF_8)] | 2025 | To estimate the overall prevalence of hepatitis B virus infection and its associated  factors among pregnant women in Ethiopia | CS = 43 | PubMed, Embase, web  of science, google scholar databases | 17,056 | Pregnant women | PROSPERO (CRD42024583487) | JBI | 6%(95% CI: 5.0, 7.0) |
| Fite et al., 2020 [[22](file:///C:\Users\abel\Desktop\PLOSE%20Hepatitis\Revision%20hepatitis\Manuscript.docx#_ENREF_22)] | 2020 | To estimate the pooled prevalence of hepatitis  B and associated risk factors in blood donors in Ethiopia. | CS = 27 | PubMed, African Journals Online (AJOL), Excerpta Medica database (EMBASE),  SCOPUS, Web of Science, Google Scholar, JSTOR | 308,188 | Blood donors | NA | NOS | 4.91% (95% CI: 4.21, 5.60) |
| Melku et al., 2021 [[23](file:///C:\Users\abel\Desktop\PLOSE%20Hepatitis\Revision%20hepatitis\Manuscript.docx#_ENREF_23)] | 2021 | Aimed at providing the pooled estimate of human immunodeficiency virus (HIV), hepatitis B virus (HBV), hepatitis C virus (HCV) and syphilis among blood donors  in Ethiopia. | PS = 17  RS = 19 | Pub-Med/Medline, EMBASE, HINARI, SCOPUS, Cochrane  database library, Google Scholar, Web of Sciences | 391,339 | Blood donors | PROSPERO (CRD42018076616) | JBI | 5.20%  (95% CI: 4.64, 5.77) |
| Gedefie et al., 2024 [[24](file:///C:\Users\abel\Desktop\PLOSE%20Hepatitis\Revision%20hepatitis\Manuscript.docx#_ENREF_24)] | 2024 | To assess the pooled prevalence of hepatitis B virus and hepatitis C virus among waste handlers in Ethiopia | CS = 8 | PubMed/crucial, Web of Science, Research Gate, Scopus | 2127 | Waste handler | PROSPERO (CRD42023398686) | JBI | 5.07% (95%CI: 2.0, 8.15) |
| EPHI and MOH (EPHI and MOH, [Unpublished]) | unpublished | Aimed to assess the prevalence of hepatitis B and C infection and associated factors among HIV infected population in Ethiopia. | CS = 12 | PubMed, Medline, Science Direct,  Google scholar | 5524 | HIV infected people | NA | DAB | 6.5% |

CS, Cross-sectional; CC, Case control; DAB, Downs and Black checklist; JBI, Joanna Briggs Institute; NA, Not available; NOS, Newcastle-Ottawa Scale; PCS-Prospective Cross sectional; PS, Prospective study; RS-Retrospective study,
