# Supplementary material for: An umbrella review navigating the nationwide burden of hepatitis B virus infection in Ethiopia: A call for action on vaccination, safe blood, and infection prevention
Source: PLoS One. 2026 Jun 22;21(6):e0352169. doi: 10.1371/journal.pone.0352169 (PMC13286280; doi:10.1371/journal.pone.0352169)
Supplement: S4 Table — (DOCX) [file pone.0352169.s004.docx]

| Authors and publication year | Item#1 | Item#2 | Item#3 | Item#4 | Item#5 | Item#6 | Item#7 | Item#8 | Item#9 | Item#10 | Item#11 | Item#12 | Item#13 | Item#14 | Item#15 | Item#16 | Overall Quality |
| --- | --- | --- | --- | --- | --- | --- | --- | --- | --- | --- | --- | --- | --- | --- | --- | --- | --- |
| Tadesse et al., 2025 [[7](#_ENREF_7)] | Y | Y | Y | Y | Y | Y | Y | Y | Y | Y | Y | Y | Y | Y | Y | Y | High |
| Yazie and Tebeje, 2019 [[12](#_ENREF_12)] | Y | Y | Y | Y | Y | Y | N | Y | Y | PY | Y | Y | Y | Y | Y | Y | moderate |
| Girmay et al., 2024 [[13](#_ENREF_13)] | Y | Y | Y | Y | Y | Y | Y | Y | Y | Y | Y | Y | Y | Y | Y | Y | High |
| Asgedom et al., 2024 [[14](#_ENREF_14)] | Y | Y | Y | Y | Y | Y | Y | Y | Y | Y | Y | Y | Y | Y | Y | Y | High |
| Kebede et al., 2018 [[15](#_ENREF_15)] | Y | N | Y | Y | Y | Y | Y | Y | Y | Y | Y | Y | Y | Y | Y | Y | Low |
| Alemu et al., 2020 [[16](#_ENREF_16)] | Y | N | Y | Y | Y | Y | PY | Y | Y | Y | Y | Y | Y | Y | Y | Y | Low |
| Belyhun et al., 2016 [[17](#_ENREF_17)] | Y | N | Y | Y | Y | Y | Y | Y | Y | Y | Y | Y | Y | Y | Y | Y | Low |
| Bitew et al., 2025 [[8](#_ENREF_8)] | Y | Y | Y | Y | Y | Y | PY | Y | Y | Y | Y | Y | Y | Y | Y | Y | High |
| Fite et al., 2020 [[18](#_ENREF_18)] | Y | N | Y | Y | Y | Y | Y | Y | Y | Y | Y | Y | Y | Y | Y | Y | Low |
| Melku et al., 2021 [[19](#_ENREF_19)] | Y | Y | Y | Y | Y | Y | Y | Y | Y | Y | Y | Y | Y | Y | Y | Y | High |
| Gedefie et al., 2024 [[20](#_ENREF_20)] | Y | Y | Y | Y | Y | Y | Y | Y | Y | Y | Y | Y | Y | Y | Y | Y | High |
| EPHI and MOH, unpublished [[21](#_ENREF_21)] | Y | N | Y | Y | Y | Y | Y | Y | Y | Y | Y | Y | Y | N | Y | Y | Low |

Y; yes; PY; partially yes; N; no; .Item#1; Did the research questions and inclusion criteria include PICO components?; Item#2; Did it contain prior protocol and the report justify any significant deviations from the protocol?; Item#3; Did the study designs for inclusion in the review was explained?; Item#4; Did a comprehensive literature search strategy was employed? Item#5; Did the review authors perform study selection in duplicate?; Item#6; Did the review authors perform data extraction in duplicate?; Item#7; Did the list of excluded studies and justification of the exclusions was provided?; Item#8; Did adequate detail of the included studies was described?; Item#9; Did the satisfactory technique for assessing the risk of bias (RoB) in individual studies that were included in the review was used?; Item#10; Did the review authors report on the sources of funding for the studies included in the review?; Item#11; If meta-analysis was performed did the review authors use appropriate methods for statistical combination of results?; Item#12; If meta-analysis was performed, did the review authors assess the potential impact of RoB in individual studies on the results of the meta-analysis or other evidence synthesis?; Item#13; Did the review authors account for RoB in individual studies when interpreting/ discussing the results of the review?; Item#14; Did the review authors provide a satisfactory explanation for, and discussion of, any heterogeneity observed in the results of the review?; Item#15; If they performed quantitative synthesis did the review authors carry out an adequate investigation of publication bias (small study bias) and discuss its likely impact on the results of the review?; Item#16; Did any potential sources of conflict of interest, including any funding they received for conducting the review was reported?
