# Supplementary material for: An umbrella review navigating the nationwide burden of hepatitis B virus infection in Ethiopia: A call for action on vaccination, safe blood, and infection prevention
Source: PLoS One. 2026 Jun 22;21(6):e0352169. doi: 10.1371/journal.pone.0352169 (PMC13286280; doi:10.1371/journal.pone.0352169)
Supplement: S5 Table — (PDF) [file pone.0352169.s005.pdf]

```
Iteration                                   Number of studies =     12
  Model: Random-effects                              observed =     12
 Method: REML                                         imputed =      0

Pooling
  Model: Random-effects
 Method: REML
```

Studies	Effect Size	[95% Conf. Interval]	
Observed	5.776	5.277	6.275
Observed + Imputed	5.776	5.277	6.275
