# Supplementary material for: An umbrella review navigating the nationwide burden of hepatitis B virus infection in Ethiopia: A call for action on vaccination, safe blood, and infection prevention
Source: PLoS One. 2026 Jun 22;21(6):e0352169. doi: 10.1371/journal.pone.0352169 (PMC13286280; doi:10.1371/journal.pone.0352169)
Supplement: Attachment — Submitted filename: Response to Reviewers.docx [file pone.0352169.s008.docx]

**Editor’s comment**

1. **Comment**: Please ensure that your manuscript meets PLOS ONE's style requirements, including those for file naming. The PLOS ONE style templates can be found at

https://journals.plos.org/plosone/s/file?id=wjVg/PLOSOne_formatting_sample_main_body.pdf and <https://journals.plos.org/plosone/s/file?id=ba62/PLOSOne_formatting_sample_title_authors_affiliations.pdf>

**Response**: Thank you for your feedback. We have thoroughly reviewed our manuscript and ensured that it meets all of PLOS ONE's style requirements, including those related to file naming conventions. We appreciate your guidance and have utilized the provided templates to align our submission accordingly.

2. **Comment**: Please amend your manuscript to include your abstract after the title page.

**Response**: Thank you— we have revised our manuscript based on the feedback, and we have added the abstract immediately after the title page

3. **Comment**: We note that you have referenced ( PHI , M., Epidemiology of Viral Hepatitis (B and C) among HIV Infected People in Ethiopia: a Systematic Review. unpublished work. ) which has currently not yet been accepted for publication. Please remove this from your References and amend this to state in the body of your manuscript: (ie “Bewick et al. [Unpublished]”) as detailed online in our guide for authors

<http://journals.plos.org/plosone/s/submission-guidelines#loc-reference-style>

**Response**: Thank you very much for highlighting this. We have corrected our manuscript by removing the citation to the unpublished work from the References section. We have also amended the in-text citation to reflect the unpublished status, now stating it as “(EPHI and MOH, [Unpublished])” in the appropriate location in the manuscript, in accordance with the PLOS ONE reference style guidance

4. **Comment**: If the reviewer comments include a recommendation to cite specific previously published works, please review and evaluate these publications to determine whether they are relevant and should be cited. There is no requirement to cite these works unless the editor has indicated otherwise.

**Response**: Thank you for your guidance. We appreciate your emphasis on the importance of relevant citations. However, we would like to clarify that the reviewer did not recommend any specific previously published works for us to cite

5. **Comment**: Please review your reference list to ensure that it is complete and correct. If you have cited papers that have been retracted, please include the rationale for doing so in the manuscript text, or remove these references and replace them with relevant current references. Any changes to the reference list should be mentioned in the rebuttal letter that accompanies your revised manuscript. If you need to cite a retracted article, indicate the article’s retracted status in the References list and also include a citation and full reference for the retraction notice

**Response**: Thank you for this request. We reviewed the reference list and confirm that it is complete and correct. In addition, no retracted papers are cited in our manuscript; therefore no changes were required.

**Reviewers comment**

**Reviewer #1**

1. **Comment**: This manuscript is a meta-analysis of previous reviews and meta-analyses of the incidence of HBsAg+ already published in other reviews and meta-analyses. I am not able to understand the utility of making a summary of previous reviews. I would say that it could be more importnat to make a meta-analysis of previous primary results, but this is my opinion.

**Response**: Thank you for your comment and concern. We agree that meta-analyses of primary studies are important; however, this manuscript is an umbrella review, which synthesizes evidence from multiple systematic reviews and meta-analyses rather than directly analyzing primary studies. Umbrella reviews are valuable when a substantial body of systematic reviews/meta-analyses exists in a given domain, because they provide a broader and more comprehensive overview of the available evidence, help clarify consistencies and discrepancies across reviews, and can support more informed conclusions about the incidence of HBsAg+ across studies. Thank you again for the suggestion. We have revised the manuscript to better clarify the rationale and utility of conducting an umbrella review for this topic, and to strengthen the justification for why synthesizing existing systematic evidence is appropriate for our research question.

2. **Comment**: The manuscript is also extremely long and complex to basically arrive at the conclusion that the rate of HBsAg+ is about 5% in Ethiopia. A simplification of the text and the figure will be appreciated by the readers.

**Response**: Thank you for your valuable feedback. We have carefully revised the text and figures to simplify the presentation and improve clarity, and we have made efforts to better align the content with the key finding that the HBsAg+ rate is approximately 5% in Ethiopia. We sincerely appreciate your recommendation.

3. **Comment**: The nomenclature used by the authors needs attention:

For example: Abstract: " Hepatitis B virus (HBV) is one of the major strains of viral hepatitis" I am sorry HBV is a virus and it is not a type ( strains) of liver disease ( hepatitis) !

**Response**: Thank you very much for this important and constructive comment. We agree that this point requires clarification, and the wording in the Abstract could be misleading. We have corrected the sentence to accurately reflect that HBV is a virus and to distinguish it from hepatitis as the disease condition. The manuscript has been revised accordingly

4. **Comment**: The authors also use the term "HBV infection" to indicate the presence of HBsAg+.

I am sorry, but an individual can be HBV infected, control the infection and become anti-HBc+, and this is a subject that was HBV infected.

HBsAg+ is the rate of HBV chronic infection not infection.

**Response**: Thank you very much for this important and constructive comment. We fully agree that our previous wording could be misleading. We have revised the manuscript to clarify the terminology and to distinguish between “HBV infection” in general and HBsAg positivity as an indicator of current hepatitis B infection status. The relevant text has been corrected accordingly.

5. **Comment**: The authors also wrote "According to the evidence synthesised from 187 countries by WHO, around 254 million people are living with hepatitis B"...

No, hepatitis B is an inflammatory liver disease caused by HBV. 254 million people are living with hepatitis B virus infection (or better use the term HBsAg+).

I recommend the authors use the terms virus, infection, and hepatitis appropriately, since this is supposed to be a scientific paper

**Response**: Thank you very much for this important and constructive comment. We agree that the terminology in the manuscript needs clarification to avoid scientific ambiguity. We have revised the sentence in the manuscript to use the correct wording and to distinguish between hepatitis B as a disease entity and HBV infection as the virologic status (HBsAg positivity) associated with the reported global estimates. The relevant text has been corrected accordingly.

**Reviewer #2**

1. **Comment**: This is an valuable umbrella study on the HBV seroprevalence in Ethiopia, yet it can be slightly improved.

**Response**: Thank you for your valuable comment and appreciation. We agree that this umbrella review provides important evidence on HBV seroprevalence in Ethiopia, and we have considered your suggestion to further improve the manuscript. We will revise the manuscript accordingly to enhance clarity and overall quality

2. **Comment**: The title is to long and You call for action in the title but you do not specify which specific public health action measures should be undertaken based on your results.

**Response**: Thank you for this important comment. We agree that the title is currently too long and that it calls for action without clearly specifying the public health measures. We have revised the title to be shorter and clearer, and we have strengthened by specifying the concrete public health actions implied by our findings. We believe these revisions address the concern and improve the manuscript’s message

3. **Comment**: In order to make the study more interesting a short introduction including Ethiopia general data should be added (population number, organisation of the health system, blood donations (voluntary? paid?), vaccination program in place?Prevention of vertical transmission in place?

**Response**: Thank you very much for your helpful comment. We have addressed this by revising the manuscript to include key Ethiopia-specific general data, such as population size, an overview of the health system organization, information on blood donation practices (voluntary vs. paid where reported), and the HBV vaccination program. We have also added details regarding prevention of vertical transmission, in line with available information. We appreciate your suggestion and believe these changes strengthen the manuscript.

4. **Comment**: The age range of the studied populations is not mentioned, I suppose those are all studies in adult populations but the age range should be mentioned.

**Response**: Thank you very much for this important comment. We agree that the age range of the included populations should be clearly stated. In our methods, we described that no age restriction was applied in the inclusion criteria. We appreciate your suggestion and believe this improves the transparency of the review.

5. **Comment**: The main risk factors (apart from sexual transmission) remain health system-associated: Blood donations, Tooth extractions, abortions-this fact needs to be commented on in more detail.

**Response**: Thank you for this important comment. We have expanded the conclusion to comment on these factors (blood transfusion and hospital admission history), and we now better interpret their potential contribution to HBV seroprevalence in Ethiopia. We excluded tooth extractions and abortions from being categorized as exclusively health system–related, since these procedures may be performed both in healthcare facilities and in the community. We appreciate your valuable input and believe the revision improves the manuscript.

6. **Comment**: The English language should be checked by a native English speaker. Avoid using the word "strain" for hepatitis B virus.

**Response**: Thank you for your valuable comment. We agree that the manuscript should be reviewed for language quality by a native English speaker. We have revised the English language throughout the manuscript to improve clarity and correctness. In addition, we have corrected the terminology and avoided using the word “strain” when referring to hepatitis B virus, and replaced it with appropriate scientific wording. We appreciate your input and believe these changes strengthen the manuscript.
