# Supplementary material for: An umbrella review navigating the nationwide burden of hepatitis B virus infection in Ethiopia: A call for action on vaccination, safe blood, and infection prevention
Source: PLoS One. 2026 Jun 22;21(6):e0352169. doi: 10.1371/journal.pone.0352169 (PMC13286280; doi:10.1371/journal.pone.0352169)
Supplement: Peer review history

# Peer review: Decision Letter 0

**DOI:** 10.1371/journal.pone.0352169.r001

**Authors:** David Chibuike Ikwuka

15 Apr 2026

PONE-D-25-65192An umbrella review of systematic reviews and meta-analysis navigating the nationwide burden of hepatitis B virus infection in Ethiopia: a call to actionPLOS One

Dear Dr. Demeke,

Thank you for submitting your manuscript to PLOS ONE. After careful consideration, we feel that it has merit but does not fully meet PLOS ONE’s publication criteria as it currently stands. Therefore, we invite you to submit a revised version of the manuscript that addresses the points raised during the review process.

Please submit your revised manuscript by May 30 2026 11:59PM. If you will need more time than this to complete your revisions, please reply to this message or contact the journal office at plosone@plos.org. When you're ready to submit your revision, log on to https://www.editorialmanager.com/pone/ and select the 'Submissions Needing Revision' folder to locate your manuscript file.

Please include the following items when submitting your revised manuscript:

- A letter that responds to each point raised by the academic editor and reviewer(s). You should upload this letter as a separate file labeled 'Response to Reviewers'.
- A marked-up copy of your manuscript that highlights changes made to the original version. You should upload this as a separate file labeled 'Revised Manuscript with Track Changes'.
- An unmarked version of your revised paper without tracked changes. You should upload this as a separate file labeled 'Manuscript'.

If you would like to make changes to your financial disclosure, please include your updated statement in your cover letter. Guidelines for resubmitting your figure files are available below the reviewer comments at the end of this letter.

If applicable, we recommend that you deposit your laboratory protocols in protocols.io to enhance the reproducibility of your results. Protocols.io assigns your protocol its own identifier (DOI) so that it can be cited independently in the future. For instructions see: https://journals.plos.org/plosone/s/submission-guidelines#loc-laboratory-protocols. Additionally, PLOS ONE offers an option for publishing peer-reviewed Lab Protocol articles, which describe protocols hosted on protocols.io. Read more information on sharing protocols at https://plos.org/protocols?utm_medium=editorial-email&utm_source=authorletters&utm_campaign=protocols.

As the corresponding author, your ORCID iD is verified in the submission system and will appear in the published article. PLOS supports the use of ORCID, and we encourage all coauthors to register for an ORCID iD and use it as well. Please encourage your coauthors to verify their ORCID iD within the submission system before final acceptance, as unverified ORCID iDs will not appear in the published article. *Only*  the individual author can complete the verification step; PLOS staff *cannot*  verify ORCID iDs on behalf of authors.

We look forward to receiving your revised manuscript.

Kind regards,

David Chibuike Ikwuka, Ph.D.

Academic Editor

PLOS One

**Journal Requirements:**

When submitting your revision, we need you to address these additional requirements.

1. Please ensure that your manuscript meets PLOS ONE's style requirements, including those for file naming. The PLOS ONE style templates can be found at

https://journals.plos.org/plosone/s/file?id=wjVg/PLOSOne_formatting_sample_main_body.pdf and

https://journals.plos.org/plosone/s/file?id=ba62/PLOSOne_formatting_sample_title_authors_affiliations.pdf

2. Please amend your manuscript to include your abstract after the title page.

3. We note that you have referenced ( PHI , M., Epidemiology of Viral Hepatitis (B and C) among HIV Infected People in Ethiopia: a Systematic Review. unpublished work. )

which has currently not yet been accepted for publication. Please remove this from your References and amend this to state in the body of your manuscript: (ie “Bewick et al. [Unpublished]”) as detailed online in our guide for authors

http://journals.plos.org/plosone/s/submission-guidelines#loc-reference-style

4. If the reviewer comments include a recommendation to cite specific previously published works, please review and evaluate these publications to determine whether they are relevant and should be cited. There is no requirement to cite these works unless the editor has indicated otherwise.

5. Please review your reference list to ensure that it is complete and correct. If you have cited papers that have been retracted, please include the rationale for doing so in the manuscript text, or remove these references and replace them with relevant current references. Any changes to the reference list should be mentioned in the rebuttal letter that accompanies your revised manuscript. If you need to cite a retracted article, indicate the article’s retracted status in the References list and also include a citation and full reference for the retraction notice.

[Note: HTML markup is below. Please do not edit.]

Reviewers' comments:

Reviewer's Responses to Questions

**Comments to the Author**

1. Is the manuscript technically sound, and do the data support the conclusions?

The manuscript must describe a technically sound piece of scientific research with data that supports the conclusions. Experiments must have been conducted rigorously, with appropriate controls, replication, and sample sizes. The conclusions must be drawn appropriately based on the data presented.

Reviewer #1: Partly

Reviewer #2: Yes

**********

2. Has the statistical analysis been performed appropriately and rigorously?

Reviewer #1: I Don't Know

Reviewer #2: Yes

**********

3. Have the authors made all data underlying the findings in their manuscript fully available?

The PLOS Data policy requires authors to make all data underlying the findings described in their manuscript fully available without restriction, with rare exception (please refer to the Data Availability Statement in the manuscript PDF file). The data should be provided as part of the manuscript or its supporting information, or deposited to a public repository. For example, in addition to summary statistics, the data points behind means, medians and variance measures should be available. If there are restrictions on publicly sharing data—e.g. participant privacy or use of data from a third party—those must be specified.

Reviewer #1: Yes

Reviewer #2: Yes

**********

4. Is the manuscript presented in an intelligible fashion and written in standard English?

PLOS ONE does not copyedit accepted manuscripts, so the language in submitted articles must be clear, correct, and unambiguous. Any typographical or grammatical errors should be corrected at revision, so please note any specific errors here.

Reviewer #1: Yes

Reviewer #2: Yes

**********

5. Review Comments to the Author

Please use the space provided to explain your answers to the questions above. You may also include additional comments for the author, including concerns about dual publication, research ethics, or publication ethics. (Please upload your review as an attachment if it exceeds 20,000 characters)

Reviewer #1: This manuscript is a meta-analysis of previous reviews and meta-analyses of the incidence of HBsAg+ already published in other reviews and meta-analyses. I am not able to understand the utility of making a summary of previous reviews. I would say that it could be more importnat to make a meta-analysis of previous primary results, but this is my opinion.

The manuscript is also extremely long and complex to basically arrive at the conclusion that the rate of HBsAg+ is about 5% in Ethiopia. A simplification of the text and the figure will be appreciated by the readers.

The nomenclature used by the authors needs attention:

For example: Abstract: " Hepatitis B virus (HBV) is one of the major strains of viral hepatitis"

I am sorry HBV is a virus and it is not a type ( strains) of liver disease ( hepatitis) !

The authors also use the term "HBV infection" to indicate the presence of HBsAg+.

I am sorry, but an individual can be HBV infected, control the infection and become anti-HBc+, and this is a subject that was HBV infected.

HBsAg+ is the rate of HBV chronic infection not infection.

The authors also wrote "According to the evidence synthesised from 187 countries by WHO, around 254 million people are living with hepatitis B"...

No, hepatitis B is an inflammatory liver disease caused by HBV. 254 million people are living with hepatitis B virus infection (or better use the term HBsAg+).

I recommend the authors use the terms virus, infection, and hepatitis appropriately, since this is supposed to be a scientific paper.

Reviewer #2: This is an valuable umbrella study on the HBV seroprevalence in Ethiopia, yet it can be slightly improved.

The title is to long.

In order to make the study more interesting a short introduction including Ethiopia general data should be added (population number, organisation of the health system, blood donations (voluntary? paid?), vaccination program in place?Prevention of vertical transmission in place?

The age range of the studied populations is not mentioned, I suppose those are all studies in adult populations but the age range should be mentioned.

The main risk factors (apart from sexual transmission) remain health system-associated: Blood donations, Tooth extractions, abortions-this fact needs to be commented on in more detail.

You call for action in the title but you do not specify which specific publichealth action measures should be undertaken based on your results.

The English language should be checked by a native English speaker. Avoid using the word "strain" for hepatitis B virus.

**********

6. PLOS authors have the option to publish the peer review history of their article (what does this mean?). If published, this will include your full peer review and any attached files.

If you choose “no”, your identity will remain anonymous but your review may still be made public.

**Do you want your identity to be public for this peer review?**  For information about this choice, including consent withdrawal, please see our Privacy Policy.

Reviewer #1: No

Reviewer #2: No

**********

[NOTE: If reviewer comments were submitted as an attachment file, they will be attached to this email and accessible via the submission site. Please log into your account, locate the manuscript record, and check for the action link "View Attachments". If this link does not appear, there are no attachment files.]

To ensure your figures meet our technical requirements, please review our figure guidelines: https://journals.plos.org/plosone/s/figures

You may also use PLOS’s free figure tool, NAAS, to help you prepare publication quality figures: https://journals.plos.org/plosone/s/figures#loc-tools-for-figure-preparation.

NAAS will assess whether your figures meet our technical requirements by comparing each figure against our figure specifications.

# Author comment: Author response to Decision Letter 1

**DOI:** 10.1371/journal.pone.0352169.r002

17 May 2026

Editor’s comment

1. Comment: Please ensure that your manuscript meets PLOS ONE's style requirements, including those for file naming. The PLOS ONE style templates can be found at

https://journals.plos.org/plosone/s/file?id=wjVg/PLOSOne_formatting_sample_main_body.pdf and https://journals.plos.org/plosone/s/file?id=ba62/PLOSOne_formatting_sample_title_authors_affiliations.pdf

Response: Thank you for your feedback. We have thoroughly reviewed our manuscript and ensured that it meets all of PLOS ONE's style requirements, including those related to file naming conventions. We appreciate your guidance and have utilized the provided templates to align our submission accordingly.

2. Comment: Please amend your manuscript to include your abstract after the title page.

Response: Thank you— we have revised our manuscript based on the feedback, and we have added the abstract immediately after the title page

3. Comment: We note that you have referenced ( PHI , M., Epidemiology of Viral Hepatitis (B and C) among HIV Infected People in Ethiopia: a Systematic Review. unpublished work. ) which has currently not yet been accepted for publication. Please remove this from your References and amend this to state in the body of your manuscript: (ie “Bewick et al. [Unpublished]”) as detailed online in our guide for authors

http://journals.plos.org/plosone/s/submission-guidelines#loc-reference-style

Response: Thank you very much for highlighting this. We have corrected our manuscript by removing the citation to the unpublished work from the References section. We have also amended the in-text citation to reflect the unpublished status, now stating it as “(EPHI and MOH, [Unpublished])” in the appropriate location in the manuscript, in accordance with the PLOS ONE reference style guidance

4. Comment: If the reviewer comments include a recommendation to cite specific previously published works, please review and evaluate these publications to determine whether they are relevant and should be cited. There is no requirement to cite these works unless the editor has indicated otherwise.

Response: Thank you for your guidance. We appreciate your emphasis on the importance of relevant citations. However, we would like to clarify that the reviewer did not recommend any specific previously published works for us to cite

5. Comment: Please review your reference list to ensure that it is complete and correct. If you have cited papers that have been retracted, please include the rationale for doing so in the manuscript text, or remove these references and replace them with relevant current references. Any changes to the reference list should be mentioned in the rebuttal letter that accompanies your revised manuscript. If you need to cite a retracted article, indicate the article’s retracted status in the References list and also include a citation and full reference for the retraction notice

Response: Thank you for this request. We reviewed the reference list and confirm that it is complete and correct. In addition, no retracted papers are cited in our manuscript; therefore no changes were required.

Reviewers comment

Reviewer #1

1. Comment: This manuscript is a meta-analysis of previous reviews and meta-analyses of the incidence of HBsAg+ already published in other reviews and meta-analyses. I am not able to understand the utility of making a summary of previous reviews. I would say that it could be more importnat to make a meta-analysis of previous primary results, but this is my opinion.

Response: Thank you for your comment and concern. We agree that meta-analyses of primary studies are important; however, this manuscript is an umbrella review, which synthesizes evidence from multiple systematic reviews and meta-analyses rather than directly analyzing primary studies. Umbrella reviews are valuable when a substantial body of systematic reviews/meta-analyses exists in a given domain, because they provide a broader and more comprehensive overview of the available evidence, help clarify consistencies and discrepancies across reviews, and can support more informed conclusions about the incidence of HBsAg+ across studies. Thank you again for the suggestion. We have revised the manuscript to better clarify the rationale and utility of conducting an umbrella review for this topic, and to strengthen the justification for why synthesizing existing systematic evidence is appropriate for our research question.

2. Comment: The manuscript is also extremely long and complex to basically arrive at the conclusion that the rate of HBsAg+ is about 5% in Ethiopia. A simplification of the text and the figure will be appreciated by the readers.

Response: Thank you for your valuable feedback. We have carefully revised the text and figures to simplify the presentation and improve clarity, and we have made efforts to better align the content with the key finding that the HBsAg+ rate is approximately 5% in Ethiopia. We sincerely appreciate your recommendation.

3. Comment: The nomenclature used by the authors needs attention:

For example: Abstract: " Hepatitis B virus (HBV) is one of the major strains of viral hepatitis" I am sorry HBV is a virus and it is not a type ( strains) of liver disease ( hepatitis) !

Response: Thank you very much for this important and constructive comment. We agree that this point requires clarification, and the wording in the Abstract could be misleading. We have corrected the sentence to accurately reflect that HBV is a virus and to distinguish it from hepatitis as the disease condition. The manuscript has been revised accordingly

4. Comment: The authors also use the term "HBV infection" to indicate the presence of HBsAg+.

I am sorry, but an individual can be HBV infected, control the infection and become anti-HBc+, and this is a subject that was HBV infected.

HBsAg+ is the rate of HBV chronic infection not infection.

Response: Thank you very much for this important and constructive comment. We fully agree that our previous wording could be misleading. We have revised the manuscript to clarify the terminology and to distinguish between “HBV infection” in general and HBsAg positivity as an indicator of current hepatitis B infection status. The relevant text has been corrected accordingly.

5. Comment: The authors also wrote "According to the evidence synthesised from 187 countries by WHO, around 254 million people are living with hepatitis B"...

No, hepatitis B is an inflammatory liver disease caused by HBV. 254 million people are living with hepatitis B virus infection (or better use the term HBsAg+).

I recommend the authors use the terms virus, infection, and hepatitis appropriately, since this is supposed to be a scientific paper

Response: Thank you very much for this important and constructive comment. We agree that the terminology in the manuscript needs clarification to avoid scientific ambiguity. We have revised the sentence in the manuscript to use the correct wording and to distinguish between hepatitis B as a disease entity and HBV infection as the virologic status (HBsAg positivity) associated with the reported global estimates. The relevant text has been corrected accordingly.

Reviewer #2

1. Comment: This is an valuable umbrella study on the HBV seroprevalence in Ethiopia, yet it can be slightly improved.

Response: Thank you for your valuable comment and appreciation. We agree that this umbrella review provides important evidence on HBV seroprevalence in Ethiopia, and we have considered your suggestion to further improve the manuscript. We will revise the manuscript accordingly to enhance clarity and overall quality

2. Comment: The title is to long and You call for action in the title but you do not specify which specific public health action measures should be undertaken based on your results.

Response: Thank you for this important comment. We agree that the title is currently too long and that it calls for action without clearly specifying the public health measures. We have revised the title to be shorter and clearer, and we have strengthened by specifying the concrete public health actions implied by our findings. We believe these revisions address the concern and improve the manuscript’s message

3. Comment: In order to make the study more interesting a short introduction including Ethiopia general data should be added (population number, organisation of the health system, blood donations (voluntary? paid?), vaccination program in place?Prevention of vertical transmission in place?

Response: Thank you very much for your helpful comment. We have addressed this by revising the manuscript to include key Ethiopia-specific general data, such as population size, an overview of the health system organization, information on blood donation practices (voluntary vs. paid where reported), and the HBV vaccination program. We have also added details regarding prevention of vertical transmission, in line with available information. We appreciate your suggestion and believe these changes strengthen the manuscript.

4. Comment: The age range of the studied populations is not mentioned, I suppose those are all studies in adult populations but the age range should be mentioned.

Response: Thank you very much for this important comment. We agree that the age range of the included populations should be clearly stated. In our methods, we described that no age restriction was applied in the inclusion criteria. We appreciate your suggestion and believe this improves the transparency of the review.

5. Comment: The main risk factors (apart from sexual transmission) remain health system-associated: Blood donations, Tooth extractions, abortions-this fact needs to be commented on in more detail.

Response: Thank you for this important comment. We have expanded the conclusion to comment on these factors (blood transfusion and hospital admission history), and we now better interpret their potential contribution to HBV seroprevalence in Ethiopia. We excluded tooth extractions and abortions from being categorized as exclusively health system–related, since these procedures may be performed both in healthcare facilities and in the community. We appreciate your valuable input and believe the revision improves the manuscript.

6. Comment: The English language should be checked by a native English speaker. Avoid using the word "strain" for hepatitis B virus.

Response: Thank you for your valuable comment. We agree that the manuscript should be reviewed for language quality by a native English speaker. We have revised the English language throughout the manuscript to improve clarity and correctness. In addition, we have corrected the terminology and avoided using the word “strain” when referring to hepatitis B virus, and replaced it with appropriate scientific wording. We appreciate your input and believe these changes strengthen the manuscript.

Attachment Submitted filename: Response to Reviewers.docx

# Editor report: Decision Letter 1

**DOI:** 10.1371/journal.pone.0352169.r003

**Authors:** David Ikwuka

7 Jun 2026

An umbrella review navigating the nationwide burden of hepatitis B virus infection in Ethiopia: a call for action on vaccination, safe blood, and infection prevention

PONE-D-25-65192R1

Dear Dr. Demeke,

We’re pleased to inform you that your manuscript has been judged scientifically suitable for publication and will be formally accepted for publication once it meets all outstanding technical requirements.

Within one week, you’ll receive an e-mail detailing the required amendments. When these have been addressed, you’ll receive a formal acceptance letter and your manuscript will be scheduled for publication.

An invoice will be generated when your article is formally accepted. Please note, if your institution has a publishing partnership with PLOS and your article meets the relevant criteria, all or part of your publication costs will be covered. Please make sure your user information is up-to-date by logging into Editorial Manager at Editorial Manager® and clicking the ‘Update My Information' link at the top of the page. For questions related to billing, please contact billing support.

If your institution or institutions have a press office, please notify them about your upcoming paper to help maximize its impact. If they’ll be preparing press materials, please inform our press team as soon as possible -- no later than 48 hours after receiving the formal acceptance. Your manuscript will remain under strict press embargo until 2 pm Eastern Time on the date of publication. For more information, please contact onepress@plos.org.

Kind regards,

David Chibuike Ikwuka, Ph.D.

Academic Editor

PLOS One

Additional Editor Comments (optional):

Reviewers' comments:

# Editor report: Acceptance letter

**DOI:** 10.1371/journal.pone.0352169.r004

**Authors:** David Ikwuka

PONE-D-25-65192R1

PLOS One

Dear Dr. Demeke,

I'm pleased to inform you that your manuscript has been deemed suitable for publication in PLOS One. Congratulations! Your manuscript is now being handed over to our production team.

At this stage, our production department will prepare your paper for publication. This includes ensuring the following:

* All references, tables, and figures are properly cited

* All relevant supporting information is included in the manuscript submission,

* There are no issues that prevent the paper from being properly typeset

You will receive further instructions from the production team, including instructions on how to review your proof when it is ready. Please keep in mind that we are working through a large volume of accepted articles, so please give us a few days to review your paper and let you know the next and final steps.

Lastly, if your institution or institutions have a press office, please let them know about your upcoming paper now to help maximize its impact. If they'll be preparing press materials, please inform our press team within the next 48 hours. Your manuscript will remain under strict press embargo until 2 pm Eastern Time on the date of publication. For more information, please contact onepress@plos.org.

You will receive an invoice from PLOS for your publication fee after your manuscript has reached the completed accept phase. If you receive an email requesting payment before acceptance or for any other service, this may be a phishing scheme. Learn how to identify phishing emails and protect your accounts at https://explore.plos.org/phishing.

If we can help with anything else, please email us at customercare@plos.org.

Thank you for submitting your work to PLOS ONE and supporting open access.

Kind regards,

PLOS ONE Editorial Office Staff

on behalf of

Dr David Chibuike Ikwuka

Academic Editor

PLOS One
